# Study on disinfection effect of a 222-nm UVC excimer lamp on object surface

**DOI:** 10.1186/s13568-023-01611-1

**Published:** 2023-09-27

**Authors:** Peiyong Ning, Yanzhen Han, Yang Liu, Shengchun Liu, Zhili Sun, Xinru Wang, Baiqi Wang, Feng Gao, Ying Wang, Yuan Wang, Xin Gao, Guanyi Chen, Xiaoyan Li

**Affiliations:** 1 Tianjin Centers for Disease Control and Prevention-Institute of Microbiology, Tianjin, 300011 China; 2 Tianjin Key Laboratory of Pathogenic Microbiology of Infectious Disease, Tianjin Centers for Disease Control and Prevention, Tianjin, 300011 China; 3 Animal, Plant and Foodstuffs Inspection Centre of Tianjin Customs, Tianjin, 300457 China; 4 Tianjin University of Commerce, Tianjin, 300134 China; 5 Department of Occupational and Environmental Health, School of Public Health, Tianjin Medical University, Tianjin, 300070 China; 6 Tianjin Key Laboratory of Environment, Nutrition and Public Health, Tianjin, 300070 China; 7 Tianjin Bureau of Commerce, Tianjin, 300040 China

**Keywords:** The 222-nm UVC excimer lamp, Surface disinfection, Standard bacteria, Cold-chain goods

## Abstract

Effective disinfection of contaminated surfaces is essential for preventing the transmission of pathogens. In this study, we investigated the UV irradiance and wavelength distribution of a 222-nm ultraviolet C (UVC) excimer lamp and its disinfection efficacy against microorganisms in laboratory conditions. By using a carrier quantitative germicidal test with stainless steel sheets as carriers, we examined the disinfection effect of the 222-nm UVC lamp on three standard strains-*Staphylococcus aureus*, *Escherichia coli*, and *Pseudomonas aeruginosa*. We tested the disinfection efficacy under different conditions by adjusting irradiation time, as well as the state and temperature of the stainless steel carriers. Our results indicated that a bacterial suspension in PBS and not-dried stainless steel carriers yielded better disinfection than in TSB and dried carriers. Additionally, carrier temperature had no significant impact on disinfection efficacy. When utilizing a bacterial suspension in PBS and non-dried carriers at a temperature of 20 °C, the three bacteria were eliminated by 222-nm UVC excimer lamp irradiation in just 15 s. In contrast, when using a bacterial suspension in TSB and dried carriers at temperatures of 20 °C, 4 °C, or − 20 °C, the three bacteria were eradicated by 222-nm UVC excimer lamp irradiation in 60 s. Comparatively, the LPM lamp required more than 10 min to achieve the same disinfection effect. Our data demonstrate that the 222-nm UVC excimer lamp has higher irradiance and a more potent microbial disinfection effect than the LPM lamp, requiring significantly less irradiation time to achieve the same disinfection effect under identical conditions. Furthermore, the 222-nm UVC excimer lamp exhibited a substantial disinfection effect on bacterial propagules at low temperatures. Our findings support the optimization of “tunnel-type” cold-chain goods disinfection devices, providing an alternative, highly efficient, and practical tool to combat the spread of SARS-CoV-2 through cold-chain systems.

**Graphical Abstract:**

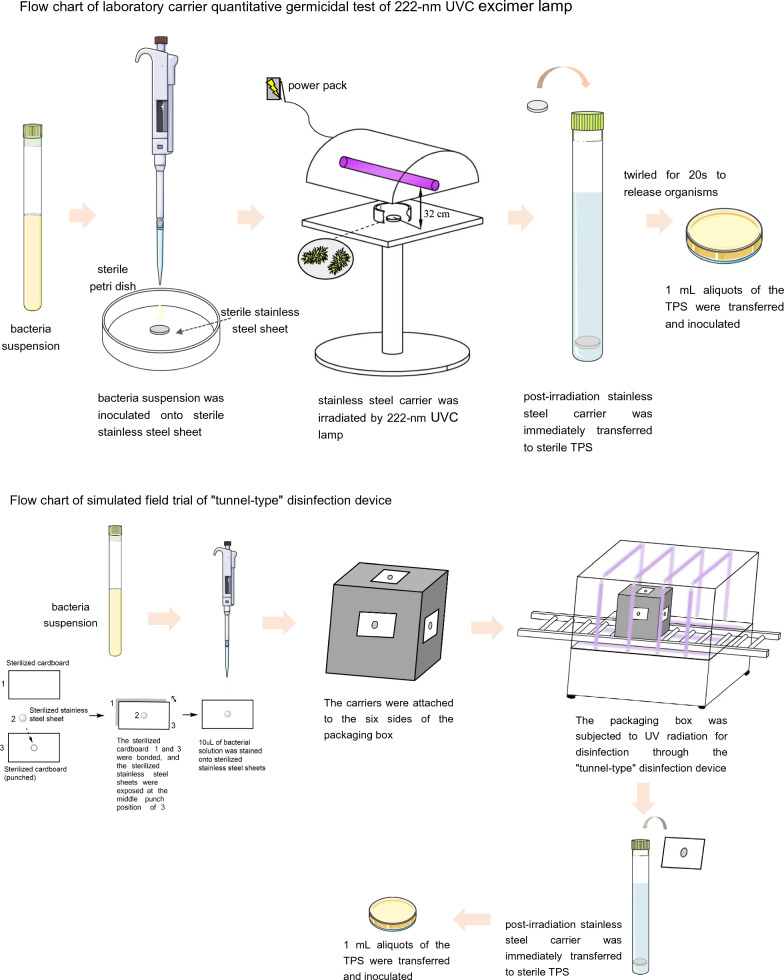

**Supplementary Information:**

The online version contains supplementary material available at 10.1186/s13568-023-01611-1.

## Key Points

- Disinfection doses of the two UV were evaluated.
- Short time irradiation by 222-nm UVC excimer lamp yielded a substantial disinfection effect on three standard strains.
- The 222-nm UVC excimer lamp contributed to a “tunnel-type” cold-chain goods disinfection equipment.

**Supplementary Information:**

The online version contains supplementary material available at 10.1186/s13568-023-01611-1.

## Introduction

Coronavirus disease 2019 (COVID-19) caused by severe acute respiratory syndrome coronavirus 2 (SARS-CoV-2) can lead to multiple organ damage or even death, making it a serious threat to human health worldwide (Raoult et al. 2020; Huang et al. 2020). It has been recognized as a public health emergency with the fastest transmission speed, the broadest range of infection, and the most challenging prevention and control in the past century (WHO 2023; Tian et al. 2021; Zhang et al. 2022). SARS-CoV-2 is primarily transmitted through respiratory droplets and close contact with infected individuals (Li et al. 2020; Liu et al. 2020a, b). However, reports have shown that object-to-human transmission of SARS-CoV-2 can occur during transnational logistics activities such as production, transportation, storage, sales, and consumption (Shao and Ye 2022). For instance, some cold chain employees have been infected with SARS-CoV-2 due to exposure to contaminated imported cold chain products (Liu et al. 2020a, b). Recent studies have indicated that SARS-CoV-2 can persist in contaminated frozen products and remain viable for several days on surfaces under controlled experimental conditions (Kampf et al. 2020; van Doremalen et al. 2020). It is also frequently detected in unopened packages and containers. Therefore, it is essential to pay close attention to the risk of infection when in contact with objects that may be contaminated with SARS-CoV-2. As globalization continues, halting international trade to control and prevent the epidemic is not a practical long-term solution. Instead, regular testing of high-risk populations and imported cold-chain products, proper disinfection of imported products, and protecting susceptible individuals while working are effective strategies for detecting and preventing the spread of SARS-CoV-2 (Chen et al. 2022). Therefore, it is crucial to cut off the transmission route of SARS-CoV-2 from objects to humans, which has become a new global challenge. At present, surface disinfection of cross-border goods is essential in inhibiting the spread of SARS-CoV-2 in the environment.

Currently, liquid chemical disinfectants are the most commonly used method to disinfect the surface of objects, which can significantly reduce the risk of contact transmission caused by packaging (Goyal et al. 2014; Godoy et al. 2021). However, disinfecting cold-chain goods, which are generally kept at low temperatures, is a special operation that differs from traditional disinfection methods. Traditional chemical disinfectants can easily freeze when sprayed on the container, making it difficult to achieve effective disinfection. Additionally, traditional disinfection methods have other drawbacks, including being time-consuming, causing secondary pollution, accumulating toxic disinfection by-products, and being tedious to perform manually (He et al. 2022; Wu et al. 2022; Benítez et al. 2021). In contrast, ultraviolet C (UVC) light-based disinfection offers several advantages, including shorter disinfection times, being safe and eco-friendly without hazardous residual, and relatively simple setup and operation, making it an alternative and reliable method (Shao and Ye 2022). Therefore, experts recommend promoting the use of green disinfection methods such as UV light during the disinfection stage of cold-chain goods and exploring strategies to improve their performance and economic benefits (He et al. 2022).

UV-irradiation is a novel technology that has gained widespread attention as a highly effective method for disinfecting surfaces from pathogenic microorganisms. It has been investigated as an alternative to conventional disinfection procedures in medical treatment, healthcare settings, and epidemic prevention (Lindsley et al. 2018; Holck et al. 2018; Tsenter et al. 2022; Yang et al. 2019). The UV spectrum can be broadly divided into four general classifications based on the wavelength's interaction with molecules: vacuum ultraviolet (VUV) with wavelengths below 200 nm, UVC with wavelengths between 200 and 280 nm, ultraviolet B (UVB) with wavelengths ranging from 280 to 315 nm, and ultraviolet A (UVA) with wavelengths ranging from 315 to 400 nm. The germicidal mechanism of UV light is primarily related to the absorption of UV by nucleic acid components. Although most of the UVB and UVA wavelengths are outside the microbial uptake peak and cannot directly kill microorganisms, UVC can cause damage to nucleic acids (DNA/RNA), primarily through the formation of thymine and pyrimidine dimers, as well as other photoproducts of nucleic acids. This damage disrupts nucleic acid replication and inactivates various pathogens (Cutler and Zimmerman 2011). Although 254-nm UVC is the most widely used germicidal wavelength, it is hazardous to human health as it can damage skin and eyes. In contrast, 222-nm UVC has gained increasing attention as a novel disinfection wavelength due to its high germicidal effectiveness and safety for human health (Narita et al. 2020; Buonanno et al. 2017; Barnard et al. 2020). Previous studies have reported that 222-nm light efficiently and safely inactivates airborne human coronaviruses, including SARS-CoV-2 (Buonanno et al. 2020; Kitagawa et al. 2021). As a result, far-UVC light (222 nm)-based disinfection systems have become increasingly visible as a reliable method for pathogen disinfection.

In this study, we aimed to evaluate the effectiveness of a 222-nm UVC excimer lamp disinfection device as a potential “tunnel-type” cold-chain goods disinfection device for surface disinfection. We tested its ability to disinfect several types of bacteria. While there are reports about the effectiveness of 222-nm UVC disinfection on SARS-CoV-2 (Buonanno et al. 2020; Kitagawa et al. 2021; Ma et al. 2021), no articles to date have investigated the application of 222-nm UVC light for surface disinfection, particularly for cold-chain goods packaging. Therefore, this study aimed to investigate the disinfection efficacy of a 222-nm UVC excimer lamp on surface contamination and lay the foundation for the development of a “tunnel-type” disinfection device that meets the following requirements: no damage to the outer packaging of cold-chain goods, no pollution to food or the environment, disinfection measures unaffected by temperature, and can achieve the disinfection effect in a very short time.

## Materials and methods

### Tested material

The 222-nm UVC excimer lamp (EX 240R10-222), half covered by an umbrella aluminum reflector, and the low pressure mercury (LPM) lamp (ZW30S19W) were kindly provided by UNILAM Co. Ltd, Korea, and Amethyst Special Light Source Co. Ltd, China, respectively. The UVC intensity was measured using an ultraviolet spectrum analyzer (OHSP-35, Hangzhou Hopoo Light & Color Technology Co. Ltd, China), which was calibrated at the factory prior to shipment. *Staphylococcus aureus* (ATCC 6538), *Escherichia coli* (8099), and *Pseudomonas aeruginosa* (ATCC 15442) were obtained from the China Center of Industrial Culture Collecting (CICC). Stainless steel sheets measuring 15 mm in diameter and 0.5 mm in thickness, which underwent degreasing treatment and autoclave sterilization prior to use, were selected as carriers. Tryptone soya broth (TSB) was purchased from OXOID Co. Ltd, and phosphate-buffered saline (PBS) was produced by Guangdong Huankai Microbial Technology Co. Ltd. The tryptone physiology solution (TPS) was composed of 1000 mg/L tryptone and 8500 mg/L sodium chloride, with a pH value of 7.0 ± 0.2.

### Preparation of microorganisms

**Fig. 1:**
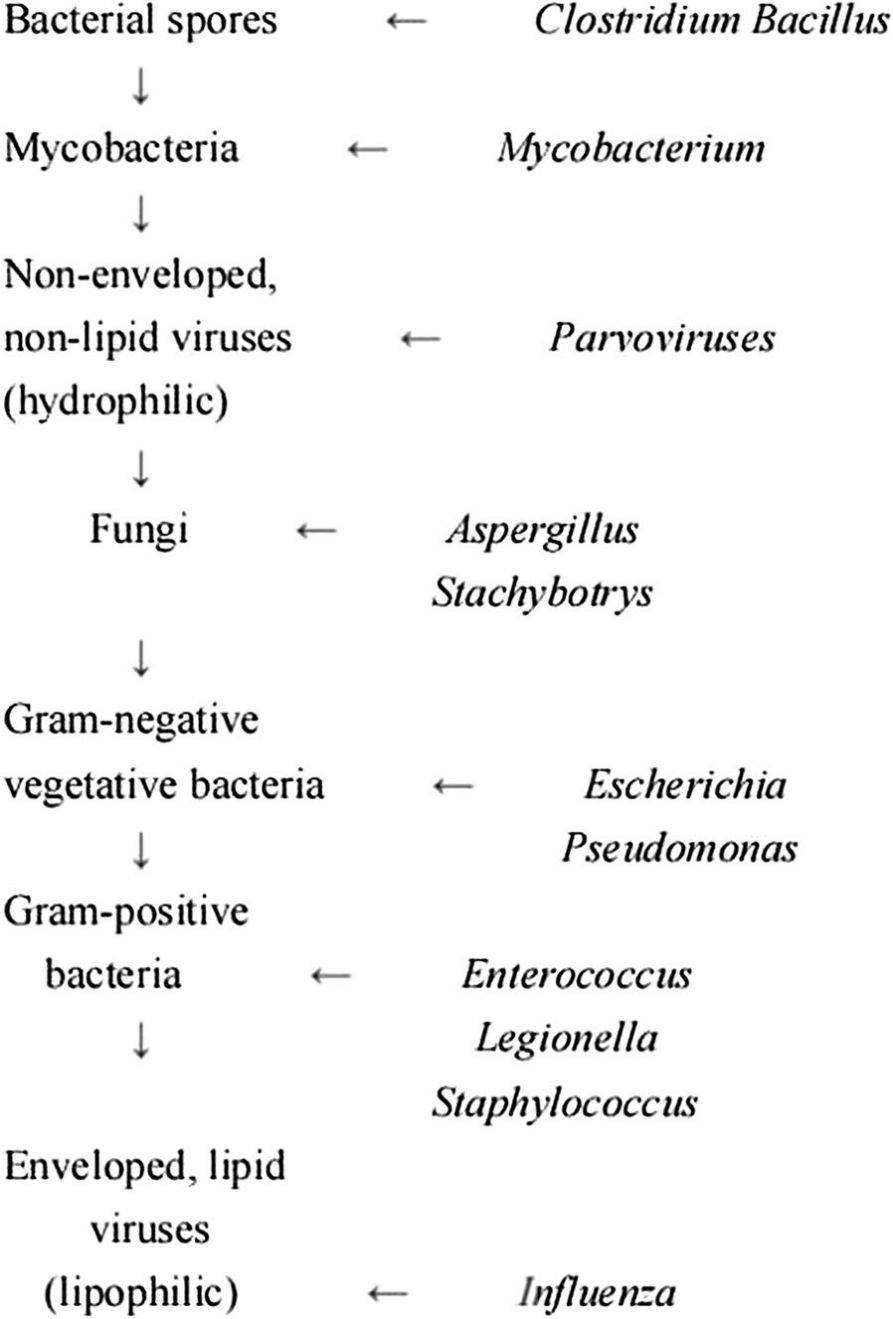
The decreasing level of resistance of microorganism to disinfection, with examples of microorganism types that are typical of each grouping

The Pathogen Detection Laboratory of Tianjin Centers for Disease Control and Prevention is a Level II Biosafety Laboratory. Given that the SARS-CoV-2 culture must be completed in a Level III Biosafety Laboratory in compliance with relevant policies, we used three standard strains—*S. aureus*, *E. coli*, and *P. aeruginosa*—to explore the disinfection effect of the 222-nm UVC excimer lamp and indirectly demonstrate the inactivation of SARS-CoV-2. Categorically, SARS-CoV-2 falls within the class of enveloped lipophilic viruses (Ijaz et al. 2022). Generally, viruses with lipid envelopes are notably more susceptible to the impact of disinfection compared with bacteria (Fig. 1) (McDonnell and Burke 2011; McDonnell 2020), we selected these bacteria to test the efficacy of the disinfection device. Fresh cultures of the three strains subcultured overnight (18–24 h) were used to prepare bacterial suspensions in both PBS solution and TSB, with a concentration of approximately 1 × 10^8^–5 × 10^8^ colony-forming units (CFU)/mL for each organism, in accordance with the Technical Standard for Disinfection (2002 Edition).

### Preparation of microorganisms stainless steel carriers, and UVC disinfection procedure

The sterile steel sheets used to monitor disinfection effectiveness were spread in a sterile petri dish. 10 μL bacterial suspensions for each organism in either PBS or TSB were inoculated onto sterile stainless steel sheets and spread to cover the entire surface using sterile inoculation loops. The prepared stainless steel carriers were allowed to dry for approximately 20 min post-inoculation and then immediately processed. The final colony count recovered from each stainless steel carrier for each bacteria should be between 5 × 10^5^–5 × 10^6^ CFU.

**Fig. 2:**
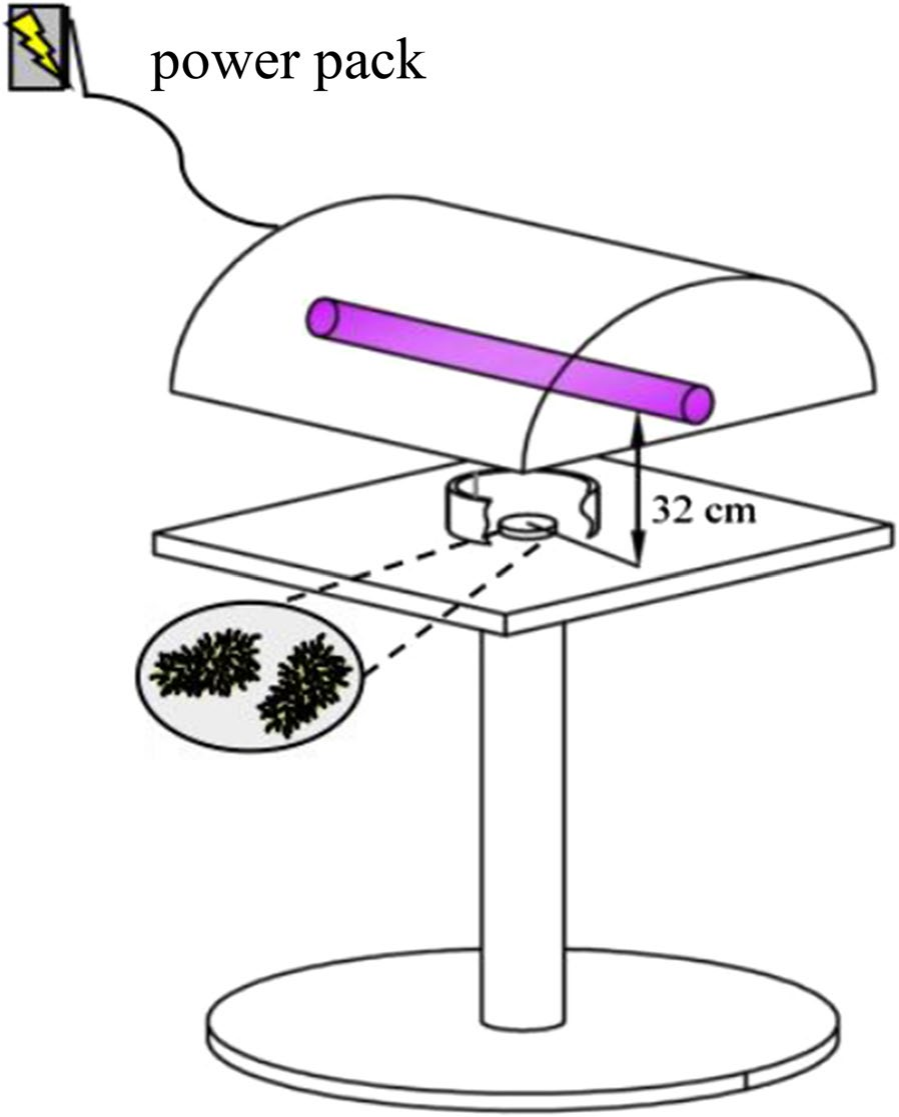
Schematic diagram of the irradiation to stainless steel carriers by the 222-nm UVC excimer lamp

Prior to each irradiation procedure, a small fan was installed to cool the lamp tube. For irradiating the stainless steel carriers, the prepared carriers were placed individually (no stacking) into a sterile petri dish. The UVC lamp was positioned directly above the center of the petri dish at a height of 32 cm above the carriers, and the lamp was supplied with 350W of power for irradiation, as shown in Fig. 2.

### Determination of the UV irradiance

Prior to the irradiation procedure, the UVC lamp was preheated for 5 min for warm-up and temperature stabilization. The irradiance at a vertical distance of 32 cm below the lamp tube was then measured using the spectrum analyzer.

### Laboratory carrier quantitative germicidal test of 222-nm UVC excimer lamp

To investigate the disinfection effect of the 222-nm UVC excimer lamp on bacterial propagules under different conditions, carrier quantitative germicidal tests were performed. The explored parameters included different irradiation times: 15, 30, 60, and 90 s, and different treatments for the preparation of stainless steel carriers: (1) bacterial suspensions prepared in PBS or TSB, (2) stainless steel carriers dried or not, and (3) dried stainless steel carriers placed at different temperatures, including traditional temperature (20 °C), preservation temperature (4 °C), or freezing temperature (− 20 °C). These different states of the treated carriers can largely represent actual objects in different states that need to be disinfected, such as dry or wet, at room temperature or low temperature. The relationship between killing log (KL) values and these various parameters could be determined.

A certain number of stainless steel carriers were prepared and subjected to UVC irradiation under different conditions as described above. After irradiation by the 222-nm UVC excimer lamp, the carriers were immediately transferred to sterile TPS and twirled for 20 s to release organisms. Then, 1 mL aliquots of the TPS solutions were transferred to nutrient agar medium and subjected to 48 h of incubation at 37 °C to assess the test group. In parallel, the same batch of carriers that were not subjected to UVC treatment were used as the positive control group. They were immediately transferred to sterile TPS solutions once the irradiance procedure for the test group was completed. The same medium (sterile TPS solution) used in this experiment was used as the negative control group. The positive and negative control groups were inoculated and cultured in the same manner. The bacteria CFUs on each plate were counted, and the total number on the control and treated stainless steel carriers was calculated. KL values were calculated by comparing log_10_ CFUs recovered from carriers after 222-nm UVC disinfection and from untreated controls, as the following:

$$ {\text{KL}} = \,{\text{mean log}}_{{{1}0}} {\text{ CFU in positive control group}} - {\text{mean log}}_{{{1}0}} {\text{ CFU in the test group}} $$

The final colony count for the recovery of each bacteria from each stainless steel carrier in the positive control group was 5 × 10^5^–5 × 10^6^ CFU (KL is 5.70–6.70), and no bacterial growth was observed in the negative control group. The disinfection was considered qualified when the KL ≥ 3. Additionally, a comparative germicidal test using the LPM lamp was conducted under the same conditions as those used for the 222-nm UVC excimer lamp.

### Simulated field trial of “tunnel-type” disinfection device

**Fig. 3:**
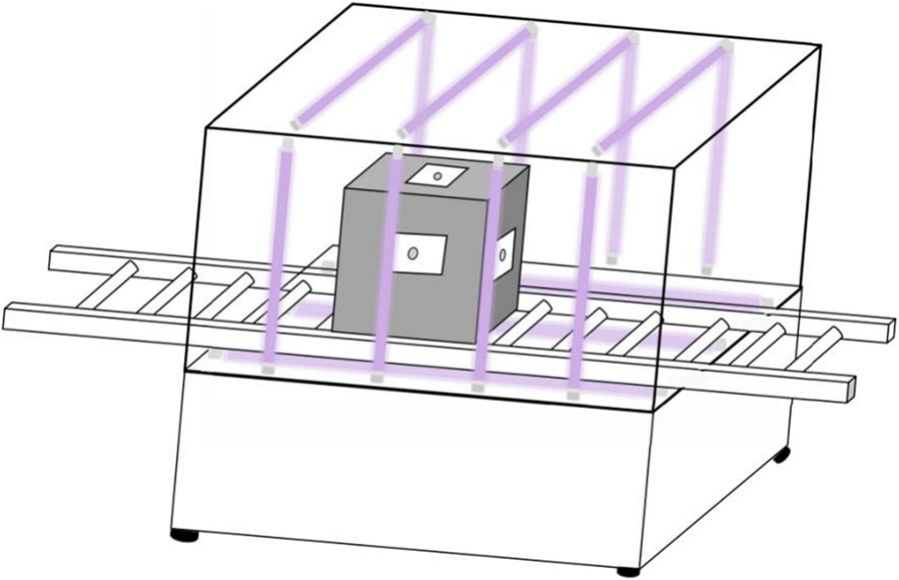
Schematic diagram of “tunnel-type” disinfection device

A total of 96 carriers were prepared for the simulated field test, with 32 for each type of bacteria (*S. aureus*, *E. coli*, *P. aeruginosa*). Among these, six carriers (two for each type) were used as controls without disinfection. The remaining carriers were arranged in groups of six and attached to the six sides of the imported cold-chain goods packing box, which was then subjected to UV radiation for disinfection through the “tunnel-type” disinfection device equipped with aluminum reflector plates on its inner walls (Fig. 3, Additional file 1: Figure S1). The exposure time of the box passing through the disinfection device was 30 s, and the distance between the six sides of the box and the ultraviolet light was kept less than 32 cm. After disinfection, the six carriers from each of the six sides of the box were collected and immediately transferred to sterile TPS, and twirled for 20 s to release organisms. Subsequently, 1 mL aliquots of the TPS solutions were transferred to nutrient agar medium and subjected to 48 h of incubation at 37 °C. The final colony count and KL values were calculated as described in “Laboratory carrier quantitative germicidal test of 222-nm UVC excimer lamp” section.

### Field test of 222-nm UVC excimer lamp

In practical applications of UV irradiation disinfection, objects that need to be disinfected (such as the surface of goods packaging) have inherent and non-artificially contaminated standard bacteria, and a variety of bacteria with varying resistance may be present. Therefore, express cartons that have been used were selected as the subject of the field test to investigate the disinfection effect of 222-nm UVC.

For the field test, 30 express cartons were collected. The opposite sides of each carton were selected randomly, and a 25 cm^2^ area was marked on each side for pre-disinfection and post-disinfection sampling. Prior to disinfection, sterile swabs pre-moistened with TPS were used to swab a 25 cm^2^ area in different directions, both horizontally and vertically, with each direction repeated eight times. The swab’s sampling end was then cut into its original TPS tube in a sterile operation. Another 25 cm^2^ area was exposed to 222-nm UVC excimer lamp irradiation for 30 s, with the lamp positioned centrally 32 cm above the area, as described in “Laboratory carrier quantitative germicidal test of 222-nm UVC excimer lamp” section. After UVC disinfection, the same approach was used to swab the 25 cm^2^ area using sterile swabs pre-moistened with TPS. The swabs’ sampling end was then cut into its original TPS tube in a sterile operation. Each swab was twirled for 20 s in 10 mL of TPS to release organisms. Subsequently, 1 mL aliquots of the TPS solutions were immediately transferred to nutrient agar medium and incubated at 37 °C for 48 h.

The control group consisted of samples from 25 cm^2^ areas that were not subjected to disinfection. The test group consisted of samples from irradiated areas of the same size that were subjected to 222-nm UVC disinfection. Negative controls were the same as those in “Laboratory carrier quantitative germicidal test of 222-nm UVC excimer lamp” section. Plate counts were conducted to estimate the total number of CFUs for all bacteria present in each sample. Finally, to calculate the KL, the disinfection is considered qualified when the KL ≥ 1.

## Results

### Determination of UV irradiance

**Fig. 4:**
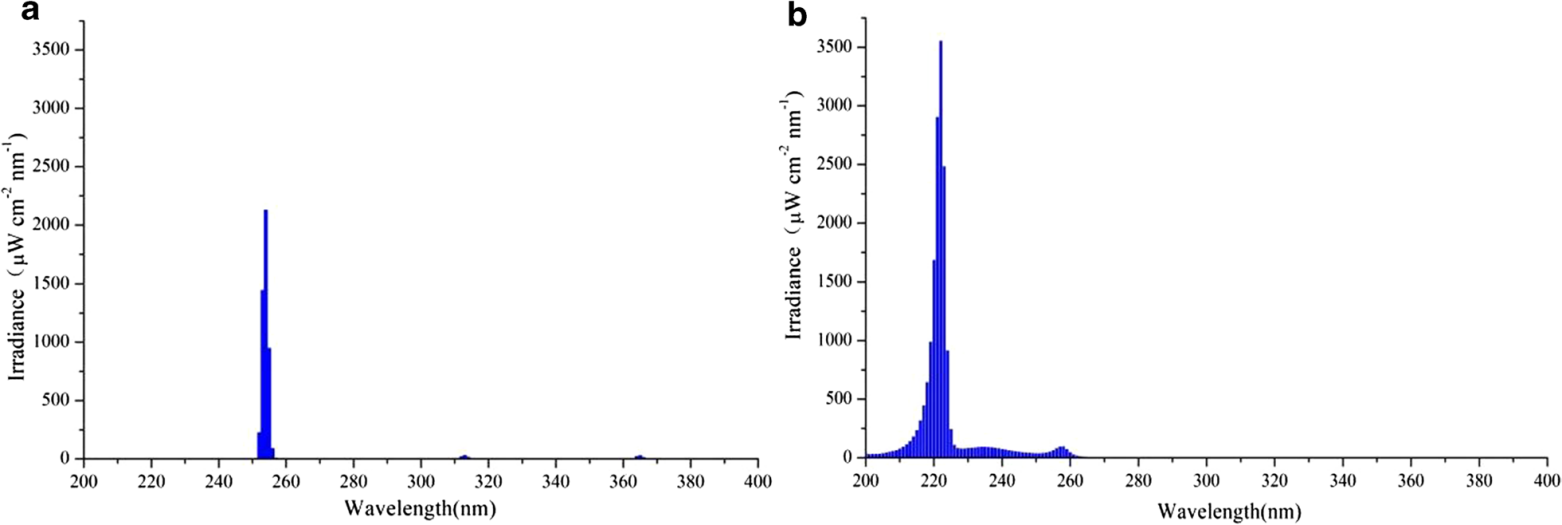
Spectrum of the emitted light intensity by LPM lamp (**a**) and 222-nm UVC excimer lamp (**b**)

The results showed that the UV irradiance of the LPM lamp and the 222-nm UVC excimer lamp at 32 cm were 484 μW/cm^2^ and 1796 μW/cm^2^, respectively. The irradiance of the 222-nm UVC excimer lamp was significantly higher than that of the LPM lamp. Additionally, the central wavelengths of the LPM lamp and the 222-nm UVC excimer lamp were 253.8 nm and 221.8 nm, respectively (Additional file 1: Table S1, Fig. 4).

### Laboratory carrier quantitative germicidal test of 222-nm UVC excimer lamp

The results showed that when a bacterial suspension prepared in sterile PBS solution was used, a good disinfection effect on bacterial propagules (KL > 3) was obtained after 27–54 mJ/cm^2^ (1796 μW/cm^2^ for 15–30 s) of 222-nm UVC irradiation. Moreover, the disinfection effect improved when no-dried carriers were used, which was achieved at a lower dose of 27 mJ/cm^2^ (1796 μW/cm^2^ for 15 s), whereas nearly double the dose (1796 μW/cm^2^ for 30 s) was required to achieve the same effect with dried carriers (Table 1). However, when a bacterial suspension prepared in TSB was used, a good disinfection effect on bacterial propagules (KL > 3) was obtained after nearly 54 mJ/cm^2^ of 222-nm UVC irradiation. Additionally, the disinfection effect improved when no-dried carriers were used, which was achieved at a dose of 54 mJ/cm^2^, whereas double the dose (1796 μW/cm^2^ for 60 s) was required to achieve the same effect with dried carriers (Table 2). These results demonstrated that the disinfection effect of the 222-nm UVC excimer lamp on bacteria in different media varied slightly. When the same disinfection effect was achieved, a higher dose was needed for bacteria in TSB, that is, longer time was needed at the same irradiance.

**Table 1:** Disinfection effect on stainless steel carriers surface by 222-nm UVC excimer lamp (temperature of the carriers was 20 °C; bacterial suspensions were prepared in PBS)

Diluent for bacterial suspension; treatment for stainless steel carriers	Mean log_10_ CFU in positive control group (range)	Mean KL ± SD for different irradiation time (s)		
		15	30	60
PBS; not dried	5.95 (5.90–5.99)	*Staphylococcus aureus*		
		3.05 ± 0.03	3.40 ± 0.05	4.06 ± 0.06
	5.91 (5.84–5.96)	*Escherichia coli*		
		3.10 ± 0.02	3.49 ± 0.03	4.18 ± 0.06
	5.87 (5.81–5.93)	*Pseudomonas aeruginosa*		
		3.09 ± 0.02	3.53 ± 0.01	4.18 ± 0.13
PBS; dried	6.02 (5.98–6.05)	*Staphylococcus aureus*		
		2.95 ± 0.01	3.21 ± 0.04	3.45 ± 0.07
	5.99 (5.95–6.03)	*Escherichia coli*		
		2.97 ± 0.01	3.25 ± 0.04	3.49 ± 0.05
	5.96 (5.90–6.03)	*Pseudomonas aeruginosa*		
		2.93 ± 0.01	3.20 ± 0.04	3.41 ± 0.05

*SD* standard deviation

**Table 2:** Disinfection effect on stainless steel carriers surface by 222-nm UVC excimer lamp (temperature of the carriers was 20 °C; bacterial suspensions were prepared in TSB)

Diluent for bacterial suspension; treatment for stainless steel carriers	Mean log_10_ CFU in positive control group (range)	Mean KL ± SD for different irradiation time (s)		
		15	30	60
TSB; not dried	6.06 (6.02–6.10)	*Staphylococcus aureus*		
		3.07 ± 0.02	3.25 ± 0.04	3.48 ± 0.02
	6.01 (5.96–6.04)	*Escherichia coli*		
		3.11 ± 0.01	3.32 ± 0.02	3.47 ± 0.06
	5.98 (5.93–6.02)	*Pseudomonas aeruginosa*		
		3.12 ± 0.03	3.34 ± 0.03	3.53 ± 0.04
TSB; dried	6.11 (6.06–6.15)	*Staphylococcus aureus*		
		2.90 ± 0.03	3.08 ± 0.02	3.31 ± 0.02
	6.08 (6.05–6.12)	*Escherichia coli*		
		2.91 ± 0.03	3.10 ± 0.01	3.36 ± 0.01
	6.04 (6.01–6.08)	*Pseudomonas aeruginosa*		
		2.92 ± 0.02	3.12 ± 0.02	3.37 ± 0.02

*SD* standard deviation

The researchers intended to design the 222-nm UVC excimer lamp as a “tunnel-type” cold-chain goods disinfection device for disinfecting the outer packaging of imported cold-chain goods, such as those stored in fresh or frozen conditions. Thus, the disinfection effect of the lamp on stainless steel carriers at different temperatures was evaluated through laboratory testing. Optimized scientific parameters would improve the conceptual popularity and microbiological effectiveness of this “tunnel-type” UVC disinfecting device for cold-chain goods. The results of this part showed that when a bacterial suspension was prepared in sterile PBS, stainless steel carriers were dried, and placed at 4 °C or − 20 °C for 2 h, the 222-nm UVC excimer lamp exerted a decent disinfection effect on bacterial propagules (KL > 3) after 54 mJ/cm^2^ (1796 μW/cm^2^ for 30 s). However, the disinfection effect was not significantly different when the carriers were at the traditional temperature of 20 °C (Table 3). Similarly, when a bacterial suspension was prepared in TSB, carriers were dried and placed at 4 °C or − 20 °C for 2 h, a good disinfection effect on bacterial propagules (KL > 3) was obtained after a double dose (1796 μW/cm^2^ for 60 s) of 222-nm UVC irradiation. However, the disinfection effect was not significantly different when the carriers were at the traditional temperature of 20 °C (Table 4).

**Table 3:** Disinfection effect on stainless steel carriers surface by 222-nm UVC excimer lamp (bacterial suspensions were prepared in PBS; stainless steel carriers were dried)

Diluent for bacterial suspension; treatment for stainless steel carriers	Temperature of the stainless steel carriers ( °C )	Mean log_10_ CFU in positive control group (range)	Mean KL ± SD for different irradiation time (s)		
			15	30	60
PBS; dried	4	6.07 (6.02–6.11)	*Staphylococcus aureus*		
			2.91 ± 0.03	3.18 ± 0.03	3.48 ± 0.03
		6.04 (6.00–6.08)	*Escherichia coli*		
			2.93 ± 0.02	3.20 ± 0.03	3.50 ± 0.03
		6.03 (5.98–6.07)	*Pseudomonas aeruginosa*		
			2.88 ± 0.04	3.16 ± 0.02	3.39 ± 0.04
	− 20	6.06 (6.01–6.10)	*Staphylococcus aureus*		
			2.88 ± 0.03	3.17 ± 0.02	3.42 ± 0.03
		6.03 (5.98–6.07)	*Escherichia coli*		
			2.89 ± 0.03	3.18 ± 0.02	3.44 ± 0.03
		6.00 (5.95–6.05)	*Pseudomonas aeruginosa*		
			2.86 ± 0.06	3.14 ± 0.02	3.42 ± 0.02

SD, standard deviation

**Table 4:** Disinfection effect on stainless steel carriers surface by 222-nm UVC excimer lamp (bacterial suspensions were prepared in TSB; stainless steel carriers were dried)

Diluent for bacterial suspension; treatment for stainless steel carriers	Temperature of the stainless steel carriers ( °C)	Mean log_10_ CFU in positive control group (range)	Mean KL ± SD for different irradiation time (s)		
			30	60	90
TSB; dried	4	6.14 (6.10–6.16)	*Staphylococcus aureus*		
			2.86 ± 0.04	3.06 ± 0.01	3.28 ± 0.03
		6.12 (6.08–6.16)	*Escherichia coli*		
			2.88 ± 0.03	3.07 ± 0.01	3.29 ± 0.02
		6.09 (6.05–6.13)	*Pseudomonas aeruginosa*		
			2.90 ± 0.02	3.08 ± 0.01	3.34 ± 0.04
	− 20	6.13 (6.09–6.16)	*Staphylococcus aureus*		
			2.83 ± 0.02	3.05 ± 0.01	3.27 ± 0.03
		6.10 (6.05–6.15)	*Escherichia coli*		
			2.87 ± 0.03	3.06 ± 0.02	3.27 ± 0.02
		6.06 (6.02–6.09)	*Pseudomonas aeruginosa*		
			2.89 ± 0.02	3.07 ± 0.03	3.33 ± 0.02

*SD* standard deviation

The results of compared germicidal test by LPM lamp showed that when a bacterial suspension was prepared in TSB, a good disinfection effect on bacterial propagules (KL > 3) was obtained after prolonged irradiation at 290.4–580.8 mJ/cm^2^ (484 μW/cm^2^ for 10–20 min) of 254-nm UV. The disinfection effect improved when no-dried carriers were used, which was achieved by irradiation at 290.4 mJ/cm^2^ (484 μW/cm^2^ for 10 min), whereas nearly double the dose (484 μW/cm^2^ for 20 min) was required to achieve the same effect with dried carriers (Table 5). These results indicate that the disinfection effect on microorganisms by the LPM lamp is much lower than that of the 222-nm UVC excimer lamp.

**Table 5:** Disinfection effect on stainless steel carriers surface by LPM lamp (temperature of the carriers was 20 °C; bacterial suspensions were prepared in TSB)

Diluent for bacterial suspension; treatment for stainless steel carriers	Mean log_10_ CFU in positive control group (range)	Mean KL ± SD for different irradiation time (min)		
		10	20	30
TSB; not dried	6.17 (6.12–6.23)	*Staphylococcus aureus*		
		3.31 ± 0.02	5.17 ± 0.25	5.77 ± 0.05
	6.12 (6.08–6.16)	*Escherichia coli*		
		3.33 ± 0.03	4.86 ± 0.20	5.62 ± 0.14
	6.11 (6.06–6.15)	*Pseudomonas aeruginosa*		
		3.39 ± 0.01	5.10 ± 0.25	5.51 ± 0.13
TSB; dried	6.37 (6.28–6.46)	*Staphylococcus aureus*		
		2.92 ± 0.03	4.29 ± 0.03	4.62 ± 0.06
	6.33 (6.23–6.41)	*Escherichia coli*		
		2.95 ± 0.02	4.33 ± 0.01	4.82 ± 0.03
	6.29 (6.16–6.40)	*Pseudomonas aeruginosa*		
		2.95 ± 0.02	4.31 ± 0.04	4.87 ± 0.26

*SD* standard deviation

### Simulated field test of “tunnel-type” disinfection device

All of the positive controls showed growth, while the negative controls did not. Upon validation of the 15 group samples, after a UVC dose of nearly 54 mJ/cm^2^ (1796 μW/cm^2^ for 30 s) using the “tunnel-type” disinfection device, the KL values for *S. aureus*, *E. coli*, and *P. aeruginosa* on those stainless steel carriers reached 3.53 (3.43–3.79), 3.63 (3.44–3.82), and 3.67 (3.55–3.83), respectively.

### Field test of 222-nm UVC excimer lamp

**Fig. 5:**
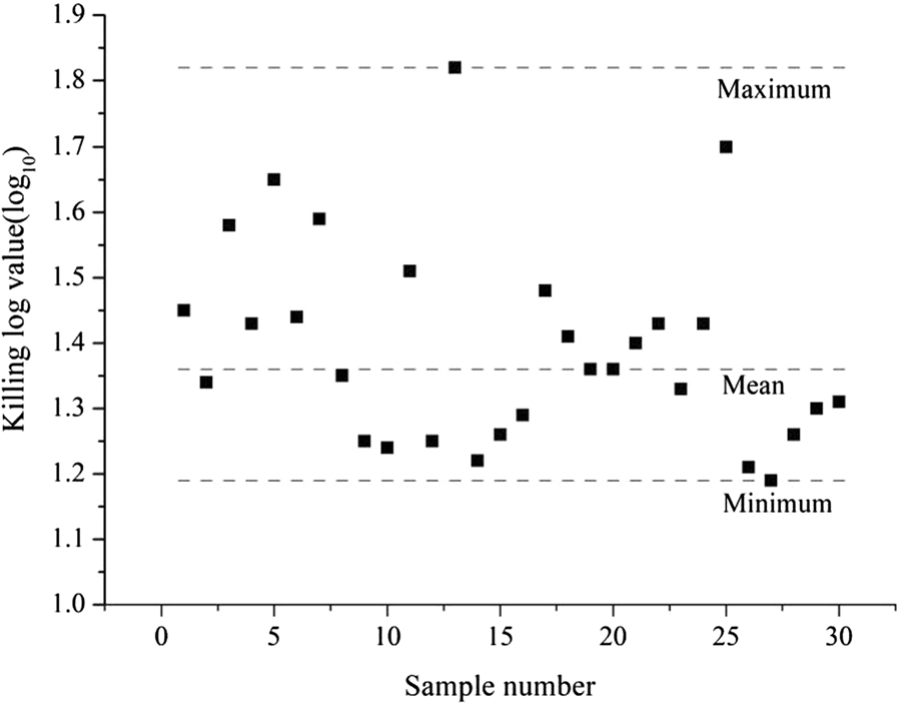
Results of field experiment

All of the samples showed growth, while the negative controls did not. Upon validation of the 30 group samples, a 222-nm UVC irradiation of nearly 54 mJ/cm^2^ (1796 μW/cm^2^ for 30 s) significantly reduced bacterial contamination on the box surface. The mean KL value was 1.36 (1.19–1.82) (Fig. 5).

## Discussion

Ultraviolet disinfection technology is a physical disinfection method that is simple and fast. A variety of disinfection devices based on the principle of ultraviolet light have been widely used for disinfection of object surfaces, air, and water treatment systems for many years. Numerous studies have verified the disinfection efficacy of UV. For instance, a study examined the effectiveness of a mobile, automatic device, the Hyper Light Disinfection Robot (model: Hyper Light P3), which utilized UVC to kill MDR-*P. aeruginosa*, MDR-*Acinetobacter baumannii*, methicillin-resistant *S. aureus* (MRSA), vancomycin-resistant *Enterococcus faecium* (VRE), *Mycobacterium abscessus*, and *Aspergillus fumigatus*. It was found that UVC irradiation of 5 min at a distance of 3 m from the device resulted in more than a 3 log_10_ reduction of vegetative bacteria colonies, except for VRE and *M. abscessus*. In uncleaned hospital rooms, significant reduction in the number of bacterial colonies sampled from different surfaces was observed after UVC irradiation for 15 min (Yang et al. 2019). In another study, disinfection tests were conducted using *Bacillus subtilis spores* as a surrogate for pathogens, and the results indicated that Ultraviolet Germicidal Irradiation (UVGI) systems can reduce microbial surface contamination in ambulance compartments (Lindsley et al. 2018). Previous research has also confirmed that both continuous UVC and pulsed UV light efficiently reduce bacterial levels on the egg surface. Furthermore, simultaneous dual-wavelength ultraviolet (DWUV) irradiation at 222 nm and 282 nm showed a decent effect in terms of a 5-log (complete) inactivation of *E. coli* and *E. faecalis* in synthetic water at pH 6.4–7.0 (Holck et al. 2018; Tsenter et al. 2022).

UV disinfection devices currently on the market include LPM lamps, pulsed xenon lamps, 222-nm UVC excimer lamps, and deep-UV LEDs, each operating at a different wavelength. The majority of UV treatment is performed with LPM lamps at 253.7 nm in medical, academic, and industrial fields. However, LPM lamps have potential drawbacks, such as the possibility of mercury leakage, a short lifetime, and significant energy requirements, which limit their application and further development (Shin et al. 2016; Tomas et al. 2022). In contrast, 222-nm UVC excimer lamps have higher irradiance and stronger microbial killing effects with shorter irradiation time, and can overcome the various inferiorities of LPM lamps, except for the need to extend the irradiation time to achieve a reliable killing effect on the surface of protein-protected and dried objects.

This study aimed to explore the disinfection effects of the 222-nm UVC excimer lamp on bacterial propagules under different conditions and compare it with the traditional LPM lamp. The results showed that a low UVC dose of 27 mJ/cm^2^ (1796 μW/cm^2^ for 15 s) was sufficient to kill *S. aureus*, *E. coli*, and *P. aeruginosa*, and KL reached 3. In our field test, a 222-nm UVC irradiation at nearly 54 mJ/cm^2^ (1796 μW/cm^2^ for 30 s) resulted in a mean KL of 1.26, demonstrating the microbiological effectiveness of the 222-nm UVC excimer lamp for surface disinfection. Good disinfection effects on bacterial propagules (KL > 3) were obtained after 54 mJ/cm^2^ of 222-nm UVC irradiation, while a prolonged irradiation at 290.4 mJ/cm^2^–580.8 mJ/cm^2^ of 254-nm UV was required. The disinfection effect of the 222-nm UVC excimer lamp is significantly stronger than that of the LPM lamp, as it requires 5–10 times less irradiance dose to achieve almost the same disinfection effect. This means that the 222-nm UVC excimer lamp achieves the same level of disinfection in a much shorter time compared to the LPM lamp. Additionally, a study found that the disinfection effect of the 222-nm UVC excimer lamp on bacteria in different media varied slightly, with slightly longer exposure times needed for bacteria in TSB. This could be attributed to the protective effect of associated proteins in TSB, which are known to pose a challenge to UVC disinfection of vegetative bacteria. These findings are consistent with previous research that highlighted the role of proteins in impeding the efficacy of UVC disinfection on bacterial cells (Wong et al. 2016). Furthermore, it was observed that the disinfection effect of the 222-nm UVC excimer lamp on non-dried carriers was slightly better than on dried ones, suggesting that the wet state of an object does not hinder the disinfection process. Additionally, there was no significant difference in the disinfection effect of the lamp on stainless steel carriers at low temperatures and at the standard temperature of 20 °C, indicating that it is effective for surface disinfection of objects at low temperatures.

Through laboratory research, we have successfully developed a “tunnel-type” disinfection device for rapid surface disinfection of imported cold-chain goods' outer packaging. Our device demonstrated a strong killing effect against standard strains, including *S. aureus*, *E. coli*, and *P. aeruginosa*. Moreover, research has shown that enveloped viruses are generally more susceptible to disinfection than most bacterial propagules. Bacterial cell walls and membranes, in contrast to viral envelopes, are indeed more resilient and robust, which can make them more resistant against disinfection. Therefore, our “tunnel-type” disinfection device is expected to be highly effective in inactivating enveloped viruses, such as SARS-CoV-2.

Our study has demonstrated the efficacy of the 222-nm UVC excimer lamp for surface disinfection, even at low temperatures. Using the hardware basis and experimental values obtained, we can optimize the scientific parameters of the “tunnel-type” cold-chain goods disinfection device, which is a popular and microbiologically effective UVC disinfecting device. The optimized device can be applied for surface disinfection of cold-chain goods and provide a highly efficient and practical tool to combat the spread of SARS-CoV-2 or other microorganisms through cold-chain systems. The “tunnel-type” disinfection device can complete the disinfection operation of imported cold-chain goods packaging within a matter of seconds, providing several advantages over chemical disinfectants, including shorter time requirements, safety and eco-friendliness, absence of hazardous residual, labor savings, and ease of operation. The application of the “tunnel-type” disinfection device has a positive impact on the speed of transfer of imported cold-chain goods, container turnover, and port storage capacity.

### Supplementary Information

**Additional file 1: Table S1.** Irradiance of LPM lamp and 222-nm UVC excimer lamp. **Figure S1.** The “tunnel-type” disinfection device.

## Data Availability

The authors agree in principle to make the data presented in the article to be available in freely accessible resources.
