# Supplementary material for: Study on disinfection effect of a 222-nm UVC excimer lamp on object surface
Source: AMB Express. 2023 Sep 27;13:102. doi: 10.1186/s13568-023-01611-1 (PMC10522550; doi:10.1186/s13568-023-01611-1)

**Additional file 1**

**Journal name: AMB Express**.

**Manuscript Title:** Study on disinfection effect of a 222-nm UVC excimer lamp on object surface.

**Authors and affiliations:**

Peiyong Ning *^a,b,1*^*, Yanzhen Han *^a,b,1^*, Yang Liu *^c^*, Shengchun Liu *^d^*, Zhili Sun *^d^*, Xinru Wang *^d^*, Baiqi Wang *^e,f^*, Feng Gao *^g^*, Ying Wang *^a,b^*, Yuan Wang *^a,b^*, Xin Gao *^a,b^*, Guanyi Chen *^d^*, Xiaoyan Li *^a,b,*^*

a.*Tianjin Centers for Disease Control and Prevention-Institute of microbiology, Tianjin 300011, China*

*b.Tianjin Key Laboratory of Pathogenic Microbiology of Infectious Disease, Tianjin Centers for Disease Control and Prevention, Tianjin 300011, China*

*c.Animal, Plant and Foodstuffs Inspection Centre of Tianjin Customs, Tianjin 300457, China*

*d.Tianjin University of Commerce, Tianjin 300134, China*

*e.Department of Occupational and Environmental Health, School of Public Health, Tianjin Medical University, Tianjin 300070, China*

*f.Tianjin Key Laboratory of Environment, Nutrition and Public Health, Tianjin 300070, China*

*g.Tianjin Bureau of Commerce, Tianjin 300040, China*

***^*^*Corresponding author**: Tianjin Centers for Disease Control and Prevention-Institute of microbiology, and Tianjin Key Laboratory of Pathogenic Microbiology of Infectious Disease, Tianjin Centers for Disease Control and Prevention, Tianjin 300011, China. E-mail address: [282536171@qq.com](mailto:282536171@qq.com) (P.Y. Ning), xiaoyanli1291@163.com (X.Y. Li); telephone and fax numbers: 8602224333601.

^1^ These authors contribute equally to this work.

**Table S1** Irradiance of LPM lamp and 222-nm UVC excimer lamp.

| Item | LPM lamp | 222-nm UVC-emitting excimer lamp |
| --- | --- | --- |
| UV hazard irradiation μw/cm^2^ | 241 | 284 |
| UVC irradiance μw/cm^2^ | 484 | 1796 |
| UVB irradiance μw/cm^2^ | 7 | 6 |
| UVA irradiance μw/cm^2^ | 6 | 20 |
| Euv irradiance μw/cm^2^ | 496 | 1822 |
| Blue light irradiance μw/cm^2^ | 0 | 0 |
| Green light irradiance μw/cm^2^ | 0 | 0 |
| Ec | 496 | 1823 |
| Irradiance Ee(μw/cm^2^) | 496 | 1823 |
| Half width(nm) | 2.1 | 3.3 |
| Peak wavelength (nm) | 253.8 | 222 |
| Central wavelength(nm) | 253.8 | 221.8 |
| Center of mass wavelength(nm) | 255.9 | 225.2 |
| Integration time(ms) | 7 | 14 |
| Peak signal | 54629 | 51655 |
| Dark signal | 2102 | 2062 |
| Compensation level | 2958 | 2958 |
| Start wavelength-stop wavelength(nm) | 200-400 | 200-400 |

**Figure S1** The "tunnel-type" disinfection device.


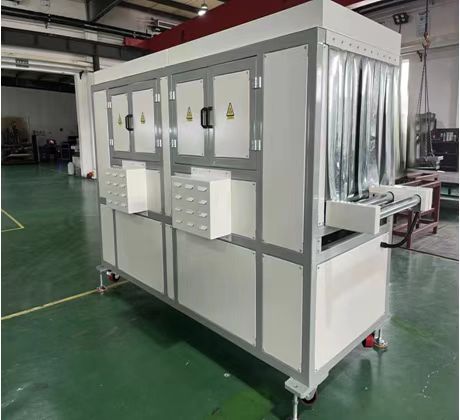

Supplement: Supplementary file 1 — Additional file 1: Table S1. Irradiance of LPM lamp and 222-nm UVC excimer lamp. Figure S1. The “tunnel-type” disinfection device. [file 13568_2023_1611_MOESM1_ESM.docx]
